# Is It Worth Domesticating?

**Published:** 1882-06

**Authors:** 


### IS IT WORTH DOMESTICATING?

M. Roland has been calling attention to an animal which is met with on the banks of South American rivers, and which he considers well suited for domestication. It is about the size of a pig, and is called the cubiaï, or the water hog. It is not an aquatic animal, but defends itself from its enemies by plunging into water and remaining there a few seconds. It lives among the reeds, and comes out morning and evening in search of its food, which consists of herbs and roots of all sorts. Its large incisors enable it to cut the hardest of woods with ease. Taken young it is easily tamed. It knows its master, and likes to be caressed, and it is very cleanly in its habits. Its skin forms supple and permeable leather, and the flesh is good eating. Without requiring more care than rabbits, it will furnish as much flesh as a sheep, though the flesh is less delicate. The apathetic character of the animal enables it to utilize all that it absorbs, so that there is no need to fatten it, and a large number may be kept in a small space. It is not afraid of cold ; in very hot weather it lies in water among the reeds. The scientific name is not given, but the animal is probably a member of the family potamochœrus which frequent swampy grounds.

